# Right Ventricular Thrombus Masquerading as a Tumor

**DOI:** 10.7759/cureus.26014

**Published:** 2022-06-16

**Authors:** Supraja Achuthanandan, Chad L Harris, Arafat A Farooqui, Gerald Hollander

**Affiliations:** 1 Internal Medicine, Maimonides Medical Center, Brooklyn, USA; 2 Cardiology, Maimonides Medical Center, Brooklyn, USA

**Keywords:** benign cardiac tumor, right ventricle, thrombus, cardiac mass, pulmonary embolism (pe), cardiac tumor in adults, cardiac myxoma, ventricular mass, cardiac thrombus, ventricular thrombus

## Abstract

Cardiac tumors are an uncommon phenomenon. Although they can be cardiac in origin, most represent a distant neoplastic growth metastasizing to the heart. Cardiac tumors can be benign or malignant. They may be symptomatic or, more commonly, found incidentally. Clinical presentation is typically related to that of dispersed neoplasm. We report a case of a 36-year-old young man with an unusually large and smooth-surfaced right ventricular mass. The patient presented to the emergency department with exertional dyspnea for two weeks. Past medical history was significant for deep venous thrombosis with non-adherence to anti-coagulation. Computerized tomographic (CT) angiography showed bilateral pulmonary emboli and a hypodense opacity in the right ventricle. A transthoracic echocardiogram showed a right ventricular non-mobile mass. The patient underwent surgical removal of the mass, which pathology demonstrated to be a thrombus. Cardiac masses can be difficult to differentiate based on imaging alone. Physicians should maintain a high index of suspicion for intracardiac thrombi as early identification and prompt treatment are imperative in improving patient outcomes.

## Introduction

Cardiac tumors are a rare occurrence. Although they can be cardiac in origin, most represent a distant neoplastic growth metastasizing to the heart. The prevalence of cardiac tumors is extremely low; 0.002%-0.3% at autopsy and 0.15% in echocardiographic series [1,2]. Common benign tumors include myxomas, lipomas, and fibromas. Primary cardiac malignancies are sporadic and are most commonly sarcomatous in origin. Cardiac tumors may be symptomatic or, more commonly, are found incidentally. Clinical presentation is typically related to that of dispersed neoplasm. Metastases to the heart are typically asymptomatic [2]. Cardiac pathologies involving valves and myocardium, such as myocardial infarction, atrial fibrillation, eosinophilic myocarditis, and rheumatic heart disease, can give rise to intra-cardiac thrombi, which can be mistaken for an intra-cardiac tumor [3]. However, intracardiac thrombi may also arise from non-cardiac causes [3,4]. Most of the time, patients with an intracardiac thrombus have an established deep vein thrombosis which in most cases is complicated by pulmonary embolism [5]. Right ventricular masses, i.e., thrombi, are extremely rare, especially when there is no co-existing left ventricular thrombus [6]. They carry significant mortality and hence require prompt treatment. We report a case of a young man with an unusually large and smooth-surfaced right ventricular mass found to be an organized thrombus with pulmonary embolization.

## Case presentation

A 36-year-old man presented to our emergency department with exertional dyspnea for two weeks. This was associated with pleuritic chest pain, fever, chills, rigors, malaise, fatigue, dizziness, and lightheadedness. Past medical history was significant for deep venous thrombosis with poor compliance to anti-coagulation. The patient denied smoking, alcohol consumption, or drug use, and there was no known family history of coagulopathies. On presentation, the patient was afebrile with a blood pressure of 90/53 mmHg, heart rate of 54 beats per minute, respiratory rate of 18 breaths per minute, and oxygen saturation of 100% on two liters nasal cannula. His physical exam was unremarkable.

**Figure 1:**
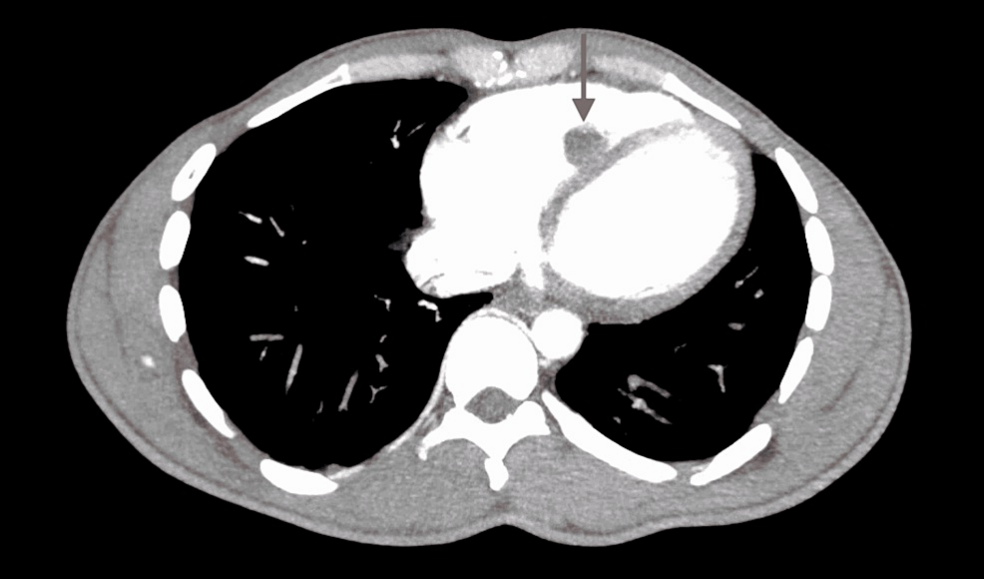
Computed tomography angiography of the chest, axial view The red arrow shows a 1.6cm rounded hypodensity within the right ventricle.

Labs demonstrated leukocytosis of 13,700/UL (4800-10,800/UL), hemoglobin 11.6 gm/dl (14-18 gm/dl), cardiac troponin 0.01 ng/ml (0.00-0.04 ng/ml), normal renal function, and unremarkable coagulation profile. Chest radiograph showed clear lungs. An electrocardiogram showed sinus rhythm with the right bundle branch block. Computerized chest tomographic (CT) angiography showed bilateral pulmonary emboli and a hypodense opacity in the right ventricle (Figure 1). A transthoracic echocardiogram showed a 4 cm x 2.8 cm right ventricular non-mobile mass attached to the right ventricular side of the interventricular septum with no definitive wall motion abnormalities (Video 1). The tricuspid valve was normal in structure with mild tricuspid regurgitation, the right atrial size was normal, and the right ventricle was both normal in size and systolic function. The left ventricle ejection fraction was estimated to be 56-60%. CT of the abdomen and pelvis with contrast did not demonstrate any evidence of renal pathology or co-existing inferior vena cava thrombus. Lower extremity Doppler ultrasonography demonstrated bilateral chronic non-occlusive thrombi.

**Video 1:** Transthoracic echocardiogram - RV-focused apical 4-chamber view 4x2.8 cm right ventricular non-mobile mass attached to the right ventricular side of the interventricular septum.

The patient was started on heparin infusion and was admitted to the cardiac care unit for further management. Cardiac magnetic resonance imaging (MRI) was ordered to characterize the mass further. However, before the MRI was performed, a multidisciplinary discussion was held about cardiothoracic surgery. The decision was made for surgical removal of the mass due to its large size and current pulmonary emboli, as non-operative treatment of a mass of this size would result in a very high risk of further embolization, cardiac arrest, and death. In the operating room, a bedside transesophageal echogram was performed to better visualize the mass prior to excision (Video 2). The procedure was performed successfully without complications, and the pathology report revealed an organizing thrombus. The patient improved significantly post-surgery and was discharged on apixaban 5 mg twice daily. The patient refused further workup for hypercoagulability on outpatient follow-up.

**Video 2:** Transesophageal echocardiogram - mid-esophageal 4-chamber view Intra-operative TEE better visualizes the RV mass adherent to the interventricular septum.

## Discussion

Various cases of ventricular masses have been reported, the most common being ventricular myxoma or thrombus. This case presented a challenge in differentiating between tumor and thrombus. This young patient presented with an unusually large and smooth-surfaced right ventricular mass without definitive wall motion abnormality. One limitation of our study is the lack of cardiac MRI, which remains the gold standard for diagnosing a cardiac myxoma. The European working group describes three types of intracardiac thrombi. Type A is highly mobile and has a serpentine shape. Type B is nearly immobile. Intermediate group thrombi are mobile but are not serpentine in shape. They have characteristics between type A and B thrombi [7]. In the present case, it was found to be an organized thrombus (non-mobile thrombus; Type B) [6,8]. As right ventricular thrombus with pulmonary embolization can be life-threatening, this case report emphasizes the high index of suspicion necessary for early diagnosis and appropriate management of the same [9-11]. This case already had very high pre-test clinical gestalt and suspicion towards a thromboembolic pathology as opposed to an RV mass (history of deep vein thrombosis, poor compliance to anti-coagulation, and absence of neoplastic systemic symptoms and signs). However, the echocardiogram findings were challenging as they deviated from the clinical judgment toward a mass.

Right ventricular thrombus formation is a relatively rare occurrence but is associated with pulmonary embolism in 4% of cases [5]. They are often associated with thromboembolic disease, hypercoagulable states, malignancy, collagen vascular diseases, amyloidosis, auto-immune diseases, endomyocardial fibrosis, and arrhythmogenic right ventricular dysplasia [6]. Although relatively rare, these thrombus formations have proven to be very deadly, with mortality rates reported around 27-45% [5]. A high index of clinical suspicion is vital to preventing disastrous consequences. On the other hand, ventricular tumors are less common. Both entities are masses within the right ventricle and can interfere with filling or outflow from the right ventricle, leading to symptoms of right-sided heart failure [12]. Symptoms include shortness of breath and syncope, such as those experienced by this patient.

The available treatment options include medical (thrombolysis, anti-coagulation), catheter-based procedures, and surgical [13]. Multivariate analyses have demonstrated that thrombolysis and surgical embolectomy have better outcomes than anti-coagulation [11,13]. Rose et al. [14] also demonstrated improvements in mortality with thrombolysis compared to anti-coagulation and surgery. However, no randomized controlled trials are available to report the superiority or inferiority of either option; hence the treatment should be individualized. Regarding our patient, a multidisciplinary discussion was held, and the decision was made for surgical intervention given the large thrombus size and high risk for further embolic events.

## Conclusions

Cardiac masses can be difficult to differentiate based on imaging alone. Physicians should maintain a high index of suspicion for intracardiac thrombi as early identification and prompt treatment are imperative in improving patient outcomes.
